# New trend of drugs abused by secondary school students in Nigeria

**DOI:** 10.4314/ahs.v21i3.57

**Published:** 2021-09

**Authors:** Ochuko E Nabofa

**Affiliations:** Department of Human Kinetics, Recreation and Sports Science Education, Delta State University, Abraka, Nigeria

**Keywords:** Adolescent Health, Drug Abuse, Drug Effects, Drug Safety, Drug Utilisation

## Abstract

**Background:**

It appears that there is a new trend in the types of drugs abused by secondary school students in Nigeria that makes it difficult to identify current drug abusers.

**Objectives:**

This study was conducted to reveal the trends with regards to the types of drugs abused by these students in the country.

**Methods:**

This is an online and desktop review of published articles about the types of drugs abused by secondary school students during the period that spanned from 2010–2020.

**Results:**

In all, 17 research reports were identified as having data on the types of drugs abused by secondary students in the Nigeria. It was found that 18 different drugs were empirically identified as being abused by secondary school students in 9 different states of Nigeria. The observed trend is that alcohol, cannabis, tobacco and cigarettes are the most abused drugs, while drugs that were least abused were cocaine, caffeine, glue, heroine, energy drinks, miraa, rohypnol and tramadol.

**Conclusion:**

It was concluded that studies of drug abuse by secondary school students in Nigeria are not yet robust enough to reveal the types of drugs that are currently being abused.

## Introduction

Drug abuse is described as the non-adaptive model of drug use with concomitant adverse health consequences that usually produce cognitive, behavioural, and psychological dysfunction problems among abusers^1^. Drug abuse negatively affects all the dimensions of health by distorting the proper functioning of the body and mind. Drug abuse is not a new phenomenon but one that is growing at an alarming rate, which nearly every country in the world, including Nigeria, have to tackle^2^. As shown in the United Nations' World Drug Report^3^, nearly one out of every 20 adults in the world, who are between the ages of 15 and 64 years, were confirmed drug abusers in 2014 resulting in over 29 million people worldwide suffering from drug abuse disorders.

The onset of drug abuse has been shown to begin during adolescence^1^. Transition from childhood to adolescence represents a delicate period during which initiating drug abuse may occur^2^. Drug abuse is forming a student sub-culture in Nigeria that can be devastating and can bring a lot of adverse effects on the national community^4^. Sloboda^5^ submitted that drug abuse is a global problem that is impacting not only individual lives but also whole communities. It is therefore necessary to study specific aspects of the problem, especially which drugs are being used and by whom, in the attempts to contain the problem. Therefore, establishing the types of drugs that are currently being abused by secondary school students would greatly assist intervention plans towards reducing the burden of drug abuse and prevention of adverse effects. It is because, drug abuse begins mostly during the adolescent years when people are still of secondary school age^6^. This review study was therefore conducted to reveal the current trends regarding the types of drugs abused by secondary school students in the Nigeria.

## Methodology

This is an online and desktop literature review research that focused on studies conducted to elicit the types of drugs abused by secondary school students in Nigeria. Every article related to types of drugs abused among secondary school students, which were conducted in Nigeria and published in domestic and international journals in the past ten (10) years, were investigated. The published research articles studied were obtained from academic/professional association journals and research periodicals, including Journal of Nigeria School Health Association (JNSHA), Journal of Nigeria Association Health Educators (JNHE), Journal of Health Promotion Research Association of Nigeria (JHEPRAN) and Journal of the Nigeria Association of Physical and Health Education, Recreation Sports and Dance (JNAPHER.SD).

The keywords used in the search included ‘types of drugs abused’ and ‘secondary school students’. The studies included must have measured drug abuse and elicited samples from only students in secondary school and have been published between January 2010 and March 2020. Out of the total of 20 articles retrieved and reviewed, three (3) were excluded because they did not contain data with regards to the types of drugs abused by secondary school students. A total of 17 papers were identified as having data on the types of drugs abused by secondary students in the country and so were used in this review. Four (4) of the 17 papers are articles that reviewed the types of drugs abused secondary school students in the country, while the remaining 12 articles reported data collected with regards to the types of drugs abused by secondary school students in Nigeria.

One study on ‘Counselling Strategies for the Prevention and Control of Drug Addiction in Enugu State’^7^ was excluded in this research. Although, the paper had a list of drugs commonly abused by secondary school students, it did not report any data to show that these were the types of drugs being currently abused by the students. The research on pattern of psychoactive substance use in the northern region of Nigeria^8^ had data on the types of drugs abused. It was, however, excluded from this study because the data collected were not from Secondary School students but from inmates of Kiru Rehabilitation Centre, Kano in the North Central Region of Nigeria. There was a study that reviewed the public health impact of substance use on adolescents in Yenagoa of Bayelsa State, Nigeria^9^. Although this article attempted to address the current trends and research related to the public health impact of substance use on adolescents in Yenagoa, it did not bring up any data on the types of drugs abused by secondary school students in Nigeria. This study was therefore excluded from this research. The research by Erumi^10^ was included in this study because the age group from which she got her data in Warri metropolis (15 – 30 years) can be found among secondary school students. Although, the study on ‘Curbing the Menace of Drug Use among Secondary School Students in Nigeria’2 did not collect any data, it was included in this study because it pointed at data collected by other researchers.

## Results

In 2010, one study^11^ found caffeine, analgesics, antimalaria, antibiotics, hypnosedatives, alcohol, tobacco, glues/organic solvents, cannabis, heroin, cocaine as the drugs abused.

In 2011 one study, the review by Pike^12^ stated that cocaine was identified by Nigeria Drug Law Enforcement Agency (NDLEA) Report as one of the drugs abused. In 2012, the under-listed three studies identified the indicated drugs as abused by secondary school students in Nigeria.

1. Nwagu's^13^ survey of the types of drugs commonly abused by secondary school students in government secondary schools in Igboetiti Local Government Area of Enugu State, identified Beer, Palm wine, Indian hemp and Glue as the drugs abused.

2. Ekpenyong^14^, in his survey of drug abuse in selected secondary institutions in Bayelsa State, South-South Nigeria, identified alcohol, cigarettes, miraa and bhang as the abused drugs abused.

3. Fareo^15^, in her review of the National Agency for Food and Drug Administration and Control (NAFDAC) Reports, found that the following classes of drugs were being abused:

a. Stimulants;

b. Hallucinogens;

c. Narcotics;

d. Sedatives;

e. Tranquilizers; and

f. Miscellaneous substances, such as glues, spot removers, tube repair, perfumes and chemicals.

In 2013, one study^16^ found analgesics, cannabis, tobacco, alcohol and sedatives to be among the drugs abused by secondary school students in Nigeria. In 2014, one study^17^ in Lagos, found analgesics, cannabis, tobacco, alcohol and sedatives to be among the drugs abused by secondary school students in Nigeria. In 2015, the underlisted two studies identified the indicated drugs as abused by secondary school students in Nigeria.

1. Erumi^10^, in her empirical survey of the prevalence of non-medical drug use among adolescents and young adults in Warri metropolis, identified alcohol and energy drinks to be among the drugs abused.

2. Dumbili^18^, in his literature review research found alcohol as one of the substances abused.

In 2016, the under-listed three studies identified the indicated drugs as abused.

1. Obiechina and Isiguzo^2^, in their literature review research, found that the following classes of drugs were being abused:

a. Nicotine, found in tar, cigars, cigarettes, tobacco and traditional snuff, is the mostly abused;

This study suggests, without any empirical evidence, that rohypnol, known as ‘roofies' is generally abused by

b. students of secondary and higher institutions in Nigeria;

c. Codeine syrup, which is usually mixed with soft drinks or garri soaked in water;

d. Stimulants such as caffeine, cocaine, nicotine and amphetamine;

e. Narcotics such as heroin, opium, morphine, tramadol, cannabis also known as pot, marijuana, hashish and bhang;

f. Depressants such as alcohol, barbiturates, tranquilizers and rohypnol;

g. Hallucinogens such as Lysergic acid diethylamide (LSD);

h. Inhalants, among which are volatile organic solvents (derived from industrial or household solvent products like paint, thinners, dry cleaning fluid spray lubricants, gasoline, kerosene, nail polish or remover, furniture polish and wax, fuel, gases, nitrites and anesthetic gases (chloroform, nitrous oxide and ether), commercial solvents like gasoline, kerosene, glue, and typewriter correction fluid among others) and household or commercial gasses and propellants like butane lighters, propane, hair and deodorant sprays, room deodorizer sprays, refrigerants sprays, ether, chloroform and halothane; and

i. Aphrodisiacs

2. Anyanwu, Ibekwe and Ojinnaka^19^ in their cross-sectional survey that employed the WHO Student Drug Use Questionnaire to study the pattern of substance abuse among adolescent secondary school students in Abakaliki found alcohol, Kolanut, Coffee, Cigarettes, Cannabis and Cocaine to be some of the drugs abused

3. Manyike, Chinawa, Chinawa, Obu, Nwokocha and Odetunde^20^, in their cross-sectional survey that utilised the WHO Student Drug Use Questionnaire to study the correlates of psycho-active substance use among boarding secondary school adolescents in Enugu, South East Nigeria, identified Kolanut, Alcohol, Coffee, Tobacco, Tranquillizers and Cannabis to be among the drugs abused.

In 2017, one study^21^ found alcohol and hot drinks, tobacco, Indian hemp or marijuana to be among the drugs abused.

In 2018, the under-listed two studies identified the indicated drugs as abused by secondary school students in Nigeria.

1. Idowu, Aremu, Olumide and Ogunlaja^22^, in their study, identified caffeine, cigarettes, cocaine, tramadol, heroin and cannabis to be among the drugs abused.

2. Amadi and Akpelu^23^, in their descriptive survey, identified alcohol, hot drinks, tobacco, Indian hemp, marijuana to be among the drugs abused.

In 2019, one study^24^ discovered that Cigarettes, Marijuana, Alcoholic beverages, Cocaine were among the drugs abused.

In 2020, one study^6^, a cross-sectional survey of factors associated with psychoactive substance use among inschool adolescents in Zaria Local Government Area, Kaduna State, Nigeria, discovered kolanut, sedatives, alcohol and tobacco to be among the drugs abused.

Table 1 show that 16 different types of drugs were empirically identified as being abused by secondary school students studied in 9 of the 36 states in Nigeria.

**Table 1:** Drugs Abused according to State of Study and Year of Publication

Year of Publication	Abused Drugs	State
2010	Caffeine, Analgesics, Antimalaria, Antibiotics, Hypnosedatives, Alcohol, Tobacco, Glues/Organic Solvents, Cannabis, Heroin, Cocaine	Lagos

2011	Nil	Nil

2012	Alcohol, Cigarettes, Miraa, Bhang	Bayelsa
	Alcohol, Indian hemp, Glue	Enugu
2013	Alcohol, Analgesics, Cannabis, Sedatives, Tobacco	Osun
2014	Alcohol, Analgesics, Cannabis, Sedatives, Tobacco	Lagos
2015	Alcohol, Energy drinks	Delta
2016	Alcohol, Cannabis, Cigarettes, Cocaine, Coffee, Kolanut	Ebonyi
	Alcohol, Cannabis, Coffee, Kolanut, Tobacco, Tranquillizers	Enugu
2017	Alcohol and hot drinks, Indian hemp or marijuana, Tobacco	Rivers
2018	Caffeine, Cannabis, Cigarettes, Cocaine, Heroin, Tramadol	Oyo
	Alcohol and hot drinks, Indian hemp or Marijuana, Tobacco	Rivers
2019	Alcoholic beverages, Cigarettes, Cocaine, Marijuana	Bayelsa
2020	Alcohol, Kolanut, Sedatives, Tobacco	Kaduna

Table 2 shows the trend of the types of drug abused by secondary school students in Nigeria. The table shows alcohol and alcoholic beverages were the most researched drug that could be abused by secondary school students.

**Table 2:** Trend of Drugs Empirically Identified as Abused according to Year of Publication

Drug	Frequency of Empirical Studies that Identified the Abused Drug in the Year										
	
	2010	2012	2013	2014	2015	2016	2017	2018	2019	2020	Total
Alcoholic	1	2	1	1	1	2	1	1	1	1	12
beverages											
Bhang	1	2	1	1	0	2	1	1	1	0	10
/Cannabis/ Indian hemp or Marijuana Tobacco	1	0	1	1	0	1	1	1	0	1	7
Cigarettes	0	1	0	0	0	1	0	1	1	0	4
Cocaine	1	0	0	0	0	1	0	1	1	0	4
Sedatives	1	0	1	1	0	0	0	0	0	1	4
Kolanut	0	0	0	0	0	2	0	0	0	1	3
Analgesics	1	0	1	1	0	0	0	0	0	0	3
Coffee	0	0	0	0	0	2	0	0	0	0	2
Caffeine	1	0	0	0	0	0	0	1	0	0	2
Glue	1	1	0	0	0	0	0	0	0	0	2
Heroin	1	0	0	0	0	0	0	1	0	0	2
Energy drinks	0	0	0	0	1	0	0	0	0	0	1
Miraa	0	1	0	0	0	0	0	0	0	0	1
Tramadol	0	0	0	0	0	0	0	1	0	0	1
Tranquillizers	0	0	0	0	0	1	0	0	0	0	1
Antimalaria	1	0	0	0	0	0	0	0	0	0	1
Antibiotics	1	0	0	0	0	0	0	0	0	0	1

## Discussion

It is discernible from Table 1 that drugs were being abused by secondary school students studied in 9 of the 36 states in Nigeria. The implication of this finding is that only nine (9) out of the 36 states of the Federal Republic of Nigeria had incidence of drug abuse among secondary school students in the last ten (10) years. This finding appears to contradict previous researchers’ claims that adolescents all over the country are abusers of drugs^25^–^28^. Oshodin^29^ had, earlier on, revealed that 85% of secondary school teenagers in Benin City were then current abusers of alcoholic beverages. About ten years later, Oshodin^30^ found out that adolescents, who are mostly secondary school students, initiate alcohol abuse during religious and ceremonial functions such as marriage ceremonies where they function as servers and tasters of alcoholic drinks. This cultural practice has not changed in Edo State, yet the state is not among the states where drug abuse was found among secondary school students in the literature in the past ten years. The finding that only nine (9) out of the 36 states of the Federal Republic of Nigeria had incidence of drug abuse among secondary school students in the last ten (10) years can, therefore, be interpreted to mean that the phenomenon of drug abuse by secondary school students in Nigeria is no longer being given the due attention it deserves by Public Health and Health Education researchers in Nigeria.

Table 2 shows that there are 18 different drugs that were empirically identified as being currently abused by secondary school students in Nigeria. There is a trend shown on the table that alcohol and alcoholic beverages appear to be the most abused drug in that it was reported by twelve (12) different researchers every one of the years under study. Alcohol was followed, in the trend of drugs abused, by Bhang /Cannabis/ Indian hemp or Marijuana. Indian hemp was found to have been abused every one of the years under review except one year, 2015, when no study reported that it was abused. The next most abused drug, tobacco, was found to have been abused by seven (7) different studies in six (6) out of the ten (10) years under study. Tobacco is followed by cigarettes, cocaine, and sedatives which were each found to have been abused by four (4) different studies in five (5) different years. The table also shows that kolanuts and analgesics were each found by three (3) different studies to have been abused in three (3) different years. Coffee, caffeine, glue and heroine were found to have been abused by two (2) different studies in two (2) different years. The least abused drugs were energy drinks, miraa, tramadol, tranquillizers, antimalarial and antibiotics, which were found to have been abused by only two (2) studies in two (2) of the ten (10) years under study.

This observed trend appears not to be telling the true story of the types of drugs being abused by secondary school students in modern day Nigeria. There are indications that students of secondary schools are abusing rohypnol and codeine syrup, which they usually mix with soft drink or garri soaked in water^2^. Cocaine has been observed to be a trending drug of abuse among adolescents in secondary school^19^. The need show off one's class of status in society has been advanced to be among the probable reasons why cocaine, tramadol and heroin are now trending as drugs of abuse among secondary school students in Oyo-State, South-West Nigeria^22^. Encomium Magazine^31^ had opined that secondary school students in Nigeria are shifting, in their types of abused drugs, towards such drugs as codeine linctus/syrup, tramadol and so on that can easily be obtained over the counter at big and small medicine stores. Some of the studies reviewed adopted the WHO Student Drug Use Questionnaire. The drugs, codeine syrups/linctus, miraa, cocaine, tramadol and heroin, rohypnol or roofies, considered to be the trending drugs of abuse among secondary school students in Nigeria are not listed in the WHO Student Drug Use Questionnaire. The observed trend that alcohol, cannabis, tobacco and cigarettes are the most abused drugs while cocaine, sedatives, kolanuts, analgesics, coffee, caffeine, glue, heroine energy drinks, miraa, tramadol, tranquillizers, antimalarial and antibiotics were the least types of drugs being abused by secondary school students in modern day Nigeria can thus be understandable. Adopting the WHO Student Drug Use Questionnaire as an instrument of studying drug abuse among secondary without modifying it to suit the Nigerian context is bound to yield faulty results. After all, newer drugs that were being abused by high school students in Iran were discovered because the WHO Student Drug Use Questionnaire was adapted to suit the local societal environment in Iran. Also, conspicuously missing from the reviewed literature is the fact there was no nationwide study data to establish the actual trend of drug abuse among secondary students in the country, Nigeria.

## Conclusion

Drug abuse among secondary school students in Nigeria is a public health problem that has not been well researched. Efforts being directed at preventing the ugly situation can only be meaningful if well researched national data on drug abuse among secondary school students in Nigeria are made available.

## Recommendations

1. It is necessary to conduct a nationwide study that will provide data on drug abuse among secondary school students in every state of Nigeria. In this manner, the types and peculiarities of drugs being abused by secondary school students in the country will be known according to each state.

2. The standardized instrument, WHO Student Drug Use Questionnaire, could be used for the nationwide study that is being recommended. The WHO Student Drug Use Questionnaire should, however, be modified to include those newer drugs of abuse, which are being suggested by opinion leaders and magazines.
